# Traditional clinical risk factors outperform disease activity and hematologic indices for FRAX hip fracture risk in rheumatoid arthritis

**DOI:** 10.3389/fimmu.2026.1762448

**Published:** 2026-03-10

**Authors:** Lina Zhang, Yuwei Wang, Xin Li, Sheng-Guang Li, Di Jin

**Affiliations:** 1 Department of Rheumatology and Immunology, Peking University International Hospital, Beijing, China; 2 Department of Emergency Internal Medicine, Weifang People’s Hospital, Weifang, Shandong, China; 3 Department of Rheumatology, Weifang People’s Hospital, Weifang, Shandong, China

**Keywords:** disease activity, FRAX, hip fracture, NLR, osteoporosis, rheumatoid arthritis, SDAI

## Abstract

**Background:**

Hip fractures are among the most devastating complications of osteoporosis, yet determinants of the Fracture Risk Assessment Tool (FRAX)–estimated 10-year hip fracture probability (FRAX-Hip) in rheumatoid arthritis (RA) remain incompletely defined. The incremental value of RA disease activity and complete blood count (CBC)–derived inflammatory indices beyond traditional FRAX clinical risk factors is uncertain.

**Objectives:**

To identify determinants of 10-year FRAX-Hip risk in RA and to compare the predictive performance and incremental value of RA disease activity indices and CBC-derived inflammatory markers.

**Methods:**

In a cross-sectional cohort of 248 RA patients undergoing dual-energy X-ray absorptiometry, we calculated femoral neck bone mineral density (BMD)–adjusted FRAX-Hip and defined high risk as FRAX-Hip ≥3%. Determinants were assessed using Firth penalized logistic regression and multivariable linear regression, and incremental value was evaluated using changes in area under the receiver operating characteristic curve (AUC), net reclassification improvement (NRI), and integrated discrimination improvement (IDI).

**Results:**

High FRAX-Hip risk was mainly driven by older age, female sex, lower body mass index, glucocorticoid exposure, and lower femoral neck BMD. Among disease activity measures, the Simplified Disease Activity Index (SDAI) provided the largest—yet modest—incremental improvement over a base clinical model (ΔAUC = 0.013; NRI = 0.903; IDI = 0.075). In contrast, CBC-derived inflammatory indices showed poor discrimination (AUC 0.46–0.62) and negligible incremental value. The clinical model explained >93% of the variance in log-transformed FRAX-Hip.

**Conclusions:**

Traditional FRAX clinical factors dominate FRAX-Hip risk estimation in RA. SDAI adds only modest incremental value, whereas CBC-derived indices do not improve risk stratification. FRAX with BMD remains a robust tool for identifying high-risk patients, underscoring the importance of optimizing age-, glucocorticoid-, and bone density–related risk factors while maintaining tight RA disease control.

## Introduction

Rheumatoid arthritis (RA) is a chronic inflammatory disease that accelerates generalized bone loss and predisposes patients to fragility fractures. Meta-analyses indicate that individuals with RA have roughly a two-fold higher risk of any fracture, including vertebral and hip fractures, compared with the general population, and osteoporosis remains highly prevalent in this population (1–3). Hip fractures are particularly devastating, being associated with substantial disability and excess mortality in RA (3, 4).

The Fracture Risk Assessment Tool (FRAX) provides 10-year probabilities of hip and major osteoporotic fractures based on age, sex, body mass index (BMI), clinical risk factors and, optionally, femoral neck bone mineral density (BMD).[5–8] FRAX is widely endorsed in guidelines and routine practice to guide decisions about further BMD testing and initiation of anti-osteoporotic therapy (5–7). In current FRAX implementations, RA is included as a dichotomous clinical risk factor; however, the algorithm does not account for RA disease activity or laboratory measures of systemic inflammation, and RA-specific risk functions are still being refined (3, 7).

Several cohort studies have evaluated FRAX-based major osteoporotic and hip fracture risk in RA, consistently showing that older age, female sex, lower BMI, glucocorticoid exposure and reduced BMD are major determinants of elevated 10-year FRAX probabilities (8–10). Some reports suggest that higher RA disease activity is associated with higher calculated FRAX risk, but these studies often focused on major osteoporotic fractures, did not formally compare different disease activity indices, and rarely assessed incremental predictive value beyond traditional FRAX clinical variables (8–10).

In parallel, simple complete blood count–derived inflammatory indices—such as neutrophil-to-lymphocyte ratio (NLR), platelet-to-lymphocyte ratio (PLR), monocyte-to-lymphocyte ratio (MLR), and composite markers including the systemic immune-inflammation index (SII), systemic inflammation response index (SIRI) and aggregate index of systemic inflammation (AISI)—have emerged as inexpensive measures of systemic inflammatory burden (11). Recent work has linked these indices to osteoporosis, low BMD and fragility fractures in general and postmenopausal populations. However, whether such hematologic indices add clinically relevant information to FRAX-based hip fracture risk estimates in RA, over and above established clinical risk factors and RA disease activity, remains unknown (11–14).

Therefore, we aimed to investigate, in a well-characterized RA cohort, the determinants of 10-year FRAX hip fracture probability (FRAX-Hip) and to compare the predictive performance of RA disease activity indices and complete blood count–derived inflammatory indices for high FRAX hip fracture risk (FRAX-Hip ≥3%). Specifically, we sought to (1) quantify the associations between traditional clinical risk factors, RA disease activity and hematologic indices with FRAX hip fracture probability; (2) compare the discriminative ability of composite RA disease activity scores versus hematologic indices; and (3) evaluate the incremental value of these inflammatory indices beyond age, sex, BMI, glucocorticoid use and femoral neck BMD for predicting high FRAX hip fracture risk in RA.

## Methods

### Study design and patients

This study was a retrospective cross-sectional analysis of patients with rheumatoid arthritis (RA) treated at a single tertiary rheumatology center. We identified all adults (age ≥18 years) with a confirmed diagnosis of RA according to American College of Rheumatology classification criteria (15) who underwent dual-energy X-ray absorptiometry (DXA) for bone mineral density (BMD) assessment during the study period. Patients were enrolled consecutively, yielding a cohort of 248 RA patients with complete data for fracture risk assessment. Key inclusion criteria were (1) established RA, and (2) availability of DXA-derived BMD together with the clinical and laboratory variables required for FRAX calculation and inflammatory indices. Patients with other metabolic bone diseases (e.g. primary hyperparathyroidism), other inflammatory arthritides, or missing key data were excluded. No imputation was performed; all analyses were conducted as complete-case analyses.

From electronic medical records we abstracted demographics (age, sex), anthropometrics (height, weight, body mass index [BMI]), RA disease duration, menopausal status, smoking history, alcohol intake, prior fragility fractures, parental hip fracture, and comorbid conditions. Metabolic/cardiovascular comorbidity was defined as the presence of hypertension, coronary artery disease, diabetes mellitus, or any combination of these diagnoses. RA-related treatment variables included current use of conventional synthetic disease-modifying antirheumatic drugs (csDMARDs; e.g. methotrexate, leflunomide), biologic or targeted synthetic DMARDs (tumor necrosis factor inhibitors, Janus kinase inhibitors), and oral glucocorticoids. Glucocorticoid exposure was defined as current or long-term oral glucocorticoid use, and doses were converted to prednisone-equivalent where applicable. Anti-osteoporotic therapy was categorized as (1) non-bisphosphonate treatment (calcium and/or active vitamin-D analogues) and (2) bisphosphonate or related agents. Menopausal status was defined as postmenopausal in women; all men and premenopausal women were classified as non-menopausal.

### Disease activity and inflammation assessment

RA disease activity was assessed at the time of DXA using standard composite indices. The 28-joint Disease Activity Score based on erythrocyte sedimentation rate (DAS28-ESR) was calculated from the 28-tender and 28-swollen joint counts, patient global assessment (visual analog scale), and ESR (16). DAS28-CRP was computed by substituting C-reactive protein (CRP) for ESR. The Clinical Disease Activity Index (CDAI) was defined as the sum of tender and swollen joint counts (28 joints) plus patient and evaluator global assessments (each on a 0–10 scale). The Simplified Disease Activity Index (SDAI) equaled CDAI plus CRP (mg/dL) (17). These indices were analyzed as continuous variables and also used to categorize patients into remission, low, moderate, or high disease activity according to established cut-offs.

Inflammatory markers were obtained from routine laboratory tests performed within the same clinical encounter. ESR and CRP were measured using standard hospital assays. Complete blood counts provided total white blood cell count and differential (neutrophils, lymphocytes, monocytes), platelet count, and hemoglobin. From these we derived hematologic inflammatory indices: neutrophil-to-lymphocyte ratio (NLR = neutrophils/lymphocytes), platelet-to-lymphocyte ratio (PLR = platelets/lymphocytes), and monocyte-to-lymphocyte ratio (MLR = monocytes/lymphocytes). We further calculated composite indices: the Systemic Immune-Inflammation Index (SII = neutrophils × platelets/lymphocytes) (18), the Systemic Inflammation Response Index (SIRI = neutrophils × monocytes/lymphocytes), and the Aggregate Index of Systemic Inflammation (AISI = neutrophils × platelets × monocytes/lymphocytes) (19). Rheumatoid factor (RF) and anti-cyclic citrullinated peptide (anti-CCP) antibodies were recorded, and “RF/CCP positive” was defined as not double-negative for both RF and anti-CCP.

### Bone mineral density measurement

All patients underwent DXA scanning of the lumbar spine and proximal femur at the study center using the same densitometer throughout the study. Femoral neck BMD (g/cm²) and T-scores were recorded, and lumbar spine and total hip BMD were also available. BMD categories (normal, osteopenia, osteoporosis) were defined according to World Health Organization T-score criteria (normal ≥ −1.0, osteopenia −2.5 < T ≥ −1.0, osteoporosis ≤ −2.5).

### FRAX hip fracture risk assessment

For each patient, the 10-year probability of hip fracture (FRAX-Hip, %) and major osteoporotic fracture (MOF, %) were calculated using the World Health Organization Fracture Risk Assessment Tool (FRAX^®^) (20), via the online calculator (version 4.x) with the country-specific model for mainland China (https://www.fraxplus.org/calculation-tool). The mainland China FRAX model was selected because all participants were Chinese residents in mainland China. The following variables were entered into FRAX: age, sex, BMI (from weight and height), personal history of prior fragility fracture, parental hip fracture, current smoking, long-term oral glucocorticoid use, presence of RA (yes for all participants), secondary osteoporosis (including premature menopause or other conditions where applicable), and alcohol intake (≥3 units/day). Femoral neck BMD (g/cm² or T-score) was included to derive BMD-adjusted fracture probabilities.

In the present analysis, FRAX-Hip (%) was the primary outcome. “High hip fracture risk” was defined *a priori* as a 10-year FRAX-Hip probability ≥3%, a commonly used intervention threshold in clinical practice. Patients were stratified into FRAX-Hip <3% and ≥3% groups for descriptive analyses. For regression analyses of continuous risk, we modeled the natural logarithm of the 10-year FRAX-Hip probability, log (FRAX-Hip%), to account for the right-skewed distribution of FRAX scores. Although MOF probabilities were calculated as part of the FRAX assessment, we did not perform a parallel determinant analysis for major osteoporotic fracture probability (MOF) in this manuscript in order to maintain focus on hip fracture risk, which has the greatest morbidity and mortality.

### Statistical analysis

Continuous variables were summarized as mean ± standard deviation (SD) or median with interquartile range, and categorical variables as counts and percentages. Between-group comparisons (FRAX-Hip <3% *vs* ≥3%) used Student’s t-test or Mann–Whitney U test for continuous variables and χ² or Fisher’s exact tests for categorical variables, as appropriate. Standardized mean differences (SMD) were additionally calculated to quantify the magnitude of between-group differences. Univariable logistic regression provided odds ratios (ORs) per 1 SD increase for continuous covariates or presence *vs* absence for categorical covariates.

### Multivariable models for high FRAX-Hip risk

To identify independent predictors of high FRAX-Hip risk (≥3%), we fitted multivariable logistic regression models with high-risk status as the dependent variable. Given the relatively low prevalence of FRAX-Hip ≥3% and the occurrence of very large coefficient estimates in standard maximum-likelihood models, we employed bias-reduced logistic regression using Firth’s penalized likelihood method to mitigate small-sample bias and potential quasi-complete separation.

Three prespecified models were examined:

- Model 1 (clinical model): age, sex, BMI, glucocorticoid use (yes/no), and femoral neck BMD.
- Model 2: Model 1 plus SDAI.
- Model 3: Model 2 plus NLR.

In these models, ORs and 95% confidence intervals (CIs) were reported per 1-year increase in age, per 1 kg/m² increase in BMI, per 0.1 g/cm² increase in BMD, per 1-point increase in SDAI, and per 1-unit increase in NLR; dichotomous predictors were coded as yes *vs* no.

### Linear regression of continuous FRAX-Hip probability

Parallel multivariable linear regression models were constructed with log (FRAX-Hip%) as the dependent variable and the same sets of predictors as in Models 1–3. Continuous predictors (age, BMI, BMD, SDAI, NLR) were standardized (z-scores) before entry so that regression coefficients (β) reflected the change in log (FRAX-Hip%) per 1 SD increase. Sex and glucocorticoid use were coded as binary variables (1 *vs* 0). Model fit was summarized with R² and adjusted R².

Model assumptions were checked using standard diagnostics: residuals versus fitted values (linearity), normal Q–Q plots of standardized residuals (normality), scale–location plots (homoscedasticity), and residuals versus leverage with Cook’s distance (influential observations). These diagnostics confirmed that the log-transformed models satisfied the key assumptions for valid inference.

### Discrimination, reclassification and clinical utility

The discriminative performance of individual inflammatory markers and prediction models for high FRAX-Hip risk (≥3%) was assessed using receiver operating characteristic (ROC) curves. We first generated ROC curves and corresponding area under the curve (AUC) values with 95% CIs for the following single predictors: ESR, CRP, DAS28-ESR, DAS28-CRP, CDAI, SDAI, and each CBC-derived index (NLR, PLR, MLR, SII, SIRI, AISI).

To evaluate the incremental predictive value of inflammatory indices, we built two sets of logistic models for FRAX-Hip ≥3% (using maximum-likelihood estimation):

- Base model A: age, sex, and BMI. Each RA disease activity index (ESR, CRP, DAS28-ESR, DAS28-CRP, CDAI, SDAI) was added individually to Base model A.
- Base model C: age, sex, BMI, CRP, and DAS28-ESR. Each CBC-derived index (NLR, PLR, MLR, SII, SIRI, AISI) was added individually to Base model C.

For each base/extended model pair we calculated AUC and the change in AUC (ΔAUC), as well as the continuous net reclassification improvement (NRI) and integrated discrimination improvement (IDI), following the method of Pencina et al. ΔAUC p values were obtained using bootstrap resampling with 2,000 iterations. NRI and IDI quantified, respectively, the proportion of individuals moving closer to their true risk category and the mean increase in predicted risk among cases *vs* controls when the new marker was added.

Model calibration for logistic models was examined using the Hosmer–Lemeshow goodness-of-fit test.

To assess potential clinical usefulness of the multivariable prediction models (Models 1–3), we performed decision curve analysis (DCA). Net benefit was plotted across threshold probabilities from 1% to 20%, comparing each model with default strategies of “treat all” and “treat none.”

All statistical analyses were conducted using IBM SPSS Statistics version 26.0 and R version 4.2.2. In R, we used the pROC package for ROC/AUC analysis, the nricens package for NRI and IDI calculations, and the ggDCA package for decision-curve analysis. Two-sided p values <0.05 were considered statistically significant. Results were reported in accordance with STROBE guidelines for observational studies.

### Ethical approval

The study protocol was reviewed and approved by the Medical Research Ethics Committee of Weifang People’s Hospital (Approval No. KYLL20251029-2). All procedures conformed to the principles of the Declaration of Helsinki and relevant local regulations. Because the study involved retrospective analysis of de-identified clinical data, the requirement for written informed consent was waived. Patient confidentiality was strictly maintained throughout data collection and analysis.

## Results

### Patient characteristics

A total of 248 patients with RA were included in the analysis (91.1% women; mean age 61.1 ± 10.9 years). Among them, 78 patients (31.5%) were classified as having high 10-year FRAX-Hip fracture risk (FRAX-Hip ≥3%), whereas 170 (68.5%) had FRAX-Hip <3%. Baseline characteristics according to FRAX-Hip category are summarized in **Table 1**.

**Table 1:** Baseline characteristics of RA patients stratified by 10-year FRAX-Hip fracture probability (<3% *vs* ≥3%).

Variable	Total (n=248)	Hip <3%	Hip ≥3%	SMD	Univariate OR (95% CI)	P
Demographics & clinical						
Sex (female)	226 (91.1%)	154 (90.6%)	72 (92.3%)	0.060	1.25 (0.47–3.32)	0.659
Age (years)	61.10 ± 10.95	56.08 ± 9.04	72.04 ± 5.32	1.980	90.75 (26.94–305.70)	<0.001
Disease duration (months)	49.59 ± 61.24	45.60 ± 50.63	58.28 ± 79.33	0.210	1.22 (0.94–1.57)	0.137
Menopausal status¹	196 (79.0%)	124 (72.9%)	72 (92.3%)	0.600	4.45 (1.81–10.94)	0.001
BMI (kg/m²)	23.97 ± 3.47	24.56 ± 3.42	22.68 ± 3.24	-0.560	0.55 (0.41–0.75)	<0.001
≥1 metabolic/CV comorbidity²	22 (8.9%)	6 (3.5%)	16 (20.5%)	0.600	7.05 (2.64–18.85)	<0.001
RF/CCP positive³	189 (76.2%)	130 (76.5%)	59 (75.6%)	-0.020	0.96 (0.51–1.79)	0.887
RA activity and routine inflammation						
ESR (mm/h)	30.01 ± 24.78	24.02 ± 20.30	43.06 ± 28.54	0.820	2.21 (1.62–3.00)	<0.001
CRP (mg/L)	10.84 ± 15.78	8.03 ± 11.00	16.96 ± 21.85	0.590	1.83 (1.32–2.53)	<0.001
DAS28-ESR	3.58 ± 1.12	3.25 ± 0.97	4.32 ± 1.07	1.060	2.87 (2.07–3.98)	<0.001
DAS28-CRP	3.72 ± 1.16	3.39 ± 1.02	4.42 ± 1.16	0.970	2.60 (1.90–3.56)	<0.001
CDAI	10.55 ± 13.10	8.45 ± 10.09	15.20 ± 17.24	0.530	1.78 (1.28–2.48)	<0.001
SDAI	11.78 ± 10.96	8.57 ± 6.97	18.78 ± 14.35	1.030	3.39 (2.29–5.03)	<0.001
CBC-derived inflammatory indices						
NLR	2.84 ± 1.98	2.80 ± 2.23	2.91 ± 1.32	0.050	1.05 (0.81–1.36)	0.709
PLR	178.04 ± 87.63	180.06 ± 87.30	173.65 ± 88.74	-0.070	0.93 (0.70–1.22)	0.593
MLR	0.30 ± 0.17	0.29 ± 0.19	0.30 ± 0.14	0.070	1.07 (0.83–1.39)	0.594
SII	717.88 ± 537.01	698.58 ± 573.78	759.95 ± 447.06	0.110	1.12 (0.86–1.44)	0.407
SIRI	1.22 ± 0.96	1.16 ± 1.01	1.36 ± 0.84	0.200	1.21 (0.93–1.57)	0.152
AISI	321.64 ± 296.15	298.04 ± 279.21	373.07 ± 326.07	0.250	1.27 (0.98–1.65)	0.070
Bone health						
T-score	-2.43 ± 1.68	-1.99 ± 1.58	-3.39 ± 1.48	-0.91	0.34 (0.24–0.50)	<0.001
Z-score	-1.17 ± 1.52	-1.05 ± 1.55	-1.43 ± 1.45	-0.25	0.78 (0.59–1.03)	0.075
BMD (g/cm²)	0.53 ± 0.17	0.56 ± 0.16	0.44 ± 0.16	-0.770	0.41 (0.29–0.58)	<0.001
History of fracture	12 (4.8%)	4 (2.4%)	8 (10.3%)	0.330	4.74 (1.38–16.26)	0.013
Medications						
Glucocorticoid exposure^4^	93 (37.5%)	43 (25.3%)	50 (64.1%)	0.780	5.27 (2.96–9.40)	<0.001
Non-bisphosphonate anti-osteoporotic therapy^5^	181 (73.0%)	117 (68.8%)	64 (82.1%)	0.300	2.07 (1.07–4.02)	0.031
Bisphosphonate therapy^6^	38 (15.3%)	17 (10.0%)	21 (26.9%)	0.470	3.32 (1.63–6.73)	<0.001

1. Menopausal status defined as postmenopausal women; all men and premenopausal women were classified as non-menopausal.

2. Metabolic/cardiovascular comorbidity included any documented hypertension, coronary artery disease, diabetes mellitus, or their combinations.

3. RF/CCP positive defined as not double-negative for RF and anti-CCP antibodies.

4. Glucocorticoid exposure defined as current or long-term oral glucocorticoid use.

5. Non-bisphosphonate anti-osteoporotic therapy included calcium, active vitamin D analogues, or their combinations.

6. Bisphosphonate therapy defined as any prescription of bisphosphonates or related agents.

Data are presented as mean ± standard deviation (SD) or n (%). Standardized mean differences (SMD) and odds ratios (OR) are calculated for the comparison between patients with FRAX-Hip <3% and those with FRAX-Hip ≥3%. ORs are derived from univariable logistic regression (per 1 SD increase in continuous variables, or presence *vs* absence for categorical variables).

Patients with FRAX-Hip ≥3% were older and had lower BMI and lower femoral neck BMD/T-score than those with FRAX-Hip <3% (**Table 1**). They also more frequently had prior fractures and glucocorticoid exposure and exhibited higher RA disease activity measures (ESR, CRP, DAS28-ESR, DAS28-CRP, CDAI, and SDAI), whereas CBC-derived inflammatory indices differed minimally between groups.

### Determinants of high FRAX hip fracture risk: penalized logistic regression

Multivariable penalized (bias-reduced) logistic regression identified traditional clinical factors as the dominant determinants of high FRAX-Hip risk (**Table 2**).

**Table 2:** Bias-reduced logistic regression models for high FRAX hip fracture risk (FRAX-Hip ≥3%).

Variable	Model 1 OR (95% CI)	*P*	Model 2 OR (95% CI)	*P*	Model 3 OR (95% CI)	*P*
Age (per 1 year)	1.73 (1.45–2.07)	<0.001	1.77 (1.46–2.14)	<0.001	1.80 (1.47–2.20)	<0.001
Female sex	20.56 (2.56–165.25)	0.004	17.51 (2.17–141.44)	0.007	18.23 (2.03–163.70)	0.010
BMI (per 1 kg/m²)	0.60 (0.48–0.77)	<0.001	0.61 (0.48–0.78)	<0.001	0.59 (0.45–0.77)	<0.001
Glucocorticoid use (yes vs no)	21.41 (5.29–86.66)	<0.001	9.40 (1.70–51.92)	0.010	11.71 (1.88–72.78)	0.008
BMD (g/cm²)	1.00 (≈1.00–1.00)*	–	1.00 (≈1.00–1.00)*	–	1.00 (≈1.00–1.00)*	–
SDAI (per 1 point)	–	–	1.06 (0.98–1.14)	0.131	1.05 (0.98–1.14)	0.176
NLR (per 1 unit)	–	–	–	–	0.61 (0.33–1.15)	0.128

The dependent variable is high FRAX hip fracture risk, defined as 10year FRAXHip probability ≥3%. Three multivariable models were fitted using biasreduced (penalized likelihood) logistic regression: Model 1 included age, sex, body mass index (BMI), glucocorticoid use, and femoral neck BMD; Model 2 additionally included the Simplified Disease Activity Index (SDAI); Model 3 additionally included the neutrophil–lymphocyte ratio (NLR). Odds ratios (OR) and 95% confidence intervals (CI) are reported per 1year increase in age, per 1 kg/m² increase in BMI, per 1point increase in SDAI, per 1unit increase in NLR, and per 0.1 g/cm² increase in BMD; dichotomous predictors are coded as yes *vs* no. BMI, body mass index; BMD, bone mineral density; SDAI, Simplified Disease Activity Index; NLR, neutrophil–lymphocyte ratio.

In Model 1, older age, female sex, lower BMI, and glucocorticoid exposure were independently associated with higher odds of FRAX-Hip ≥3% (**Table 2**). After adjustment for these clinical factors, femoral neck BMD was not an independent predictor.

Adding SDAI to the clinical model (Model 2) did not materially change the estimates for age, BMI, or glucocorticoid use (**Table 2**). SDAI showed a small, non-significant association with high FRAX-Hip risk, and further inclusion of the neutrophil–lymphocyte ratio (NLR) (Model 3) did not improve model performance; NLR was not independently associated with FRAX-Hip ≥3%.

### Linear regression models for continuous FRAX-Hip probability

Results of the linear regression models with log-transformed 10-year FRAX-Hip probability as the dependent variable are shown in **Table 3**. Across Models 1–3, higher age, female sex, lower BMI, and glucocorticoid exposure were strongly and consistently associated with higher log (FRAX-Hip%), whereas femoral neck BMD had a small and non-significant effect after adjustment. SDAI and NLR were not independently associated with log (FRAX-Hip%) once traditional clinical factors were considered (**Table 3**).

**Table 3:** Multivariable linear regression models for log-transformed 10-year FRAX-Hip fracture probability.

Variable	Model 1 β (95% CI)	*P*	Model 2 β (95% CI)	*P*	Model 3 β (95% CI)	*P*
Age (per 1 SD)	1.03 (0.99, 1.08)	<0.001	1.03 (0.98, 1.08)	<0.001	1.03 (0.98, 1.08)	<0.001
Female sex (vs male)	0.42 (0.25, 0.58)	<0.001	0.40 (0.23, 0.57)	<0.001	0.39 (0.23, 0.56)	<0.001
BMI (per 1 SD)	–0.24 (–0.29, –0.20)	<0.001	–0.24 (–0.28, –0.19)	<0.001	–0.24 (–0.28, –0.19)	<0.001
Glucocorticoid use (yes vs no)	0.72 (0.63, 0.81)	<0.001	0.67 (0.55, 0.79)	<0.001	0.67 (0.55, 0.79)	<0.001
BMD (per 1 SD)	–0.03 (–0.09, 0.02)	0.208	–0.03 (–0.09, 0.02)	0.188	–0.04 (–0.09, 0.02)	0.166
SDAI (per 1 SD)	–	–	0.04 (–0.02, 0.10)	0.182	0.04 (–0.02, 0.10)	0.168
NLR (per 1 SD)	–	–	–	–	–0.02 (–0.06, 0.03)	0.438
R²	0.931		0.931		0.932	–
Adjusted R²	0.929		0.93		0.93	–

The dependent variable is the natural logarithm of the 10-year FRAX-Hip (log [FRAX-Hip%]). Models 1–3 include the same sets of predictors as in **Table 2**. Continuous predictors (age, BMI, BMD, SDAI, NLR) were standardized (z-scores); regression coefficients (β) represent the change in log (FRAX-Hip%) per 1 standard deviation increase in each predictor. Categorical variables (sex, glucocorticoid use) are coded as 1 *vs* 0. R² and adjusted R² are reported for overall model fit.

**Figure 1:**
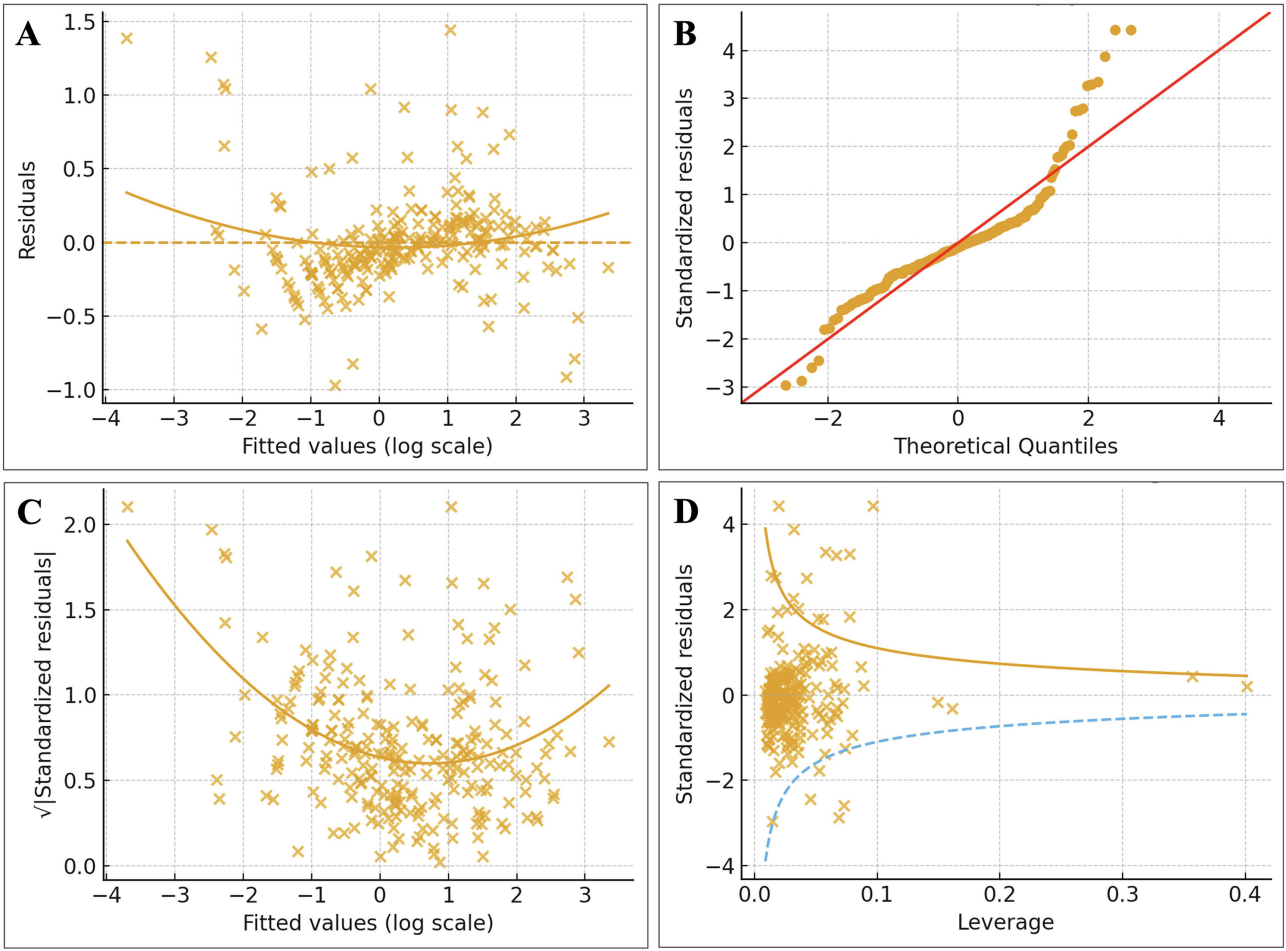
Diagnostic plots for the multivariable linear regression model predicting log-transformed FRAX hip fracture probability. This composite figure displays four standard diagnostic assessments of the final linear regression model with log-transformed FRAX-Hip probability as the dependent variable. **(A)** Residuals *vs* fitted values: residuals are randomly scattered around the horizontal axis without clear pattern, indicating that the log-transformation adequately addressed non-linearity and satisfied the linearity assumption. **(B)** Normal Q–Q plot: standardized residuals align closely with the 45-degree reference line, suggesting that the errors are approximately normally distributed. **(C)** Scale–location plot: the smoothing line is nearly horizontal across the range of fitted values, supporting homoscedasticity (constant variance of residuals). **(D)** Residuals *vs* leverage: all observations lie well within the Cook’s distance warning boundaries, indicating the absence of influential outliers. Together, these diagnostics confirm that the log-transformed linear model meets key assumptions for valid inference.

**Figure 2:**
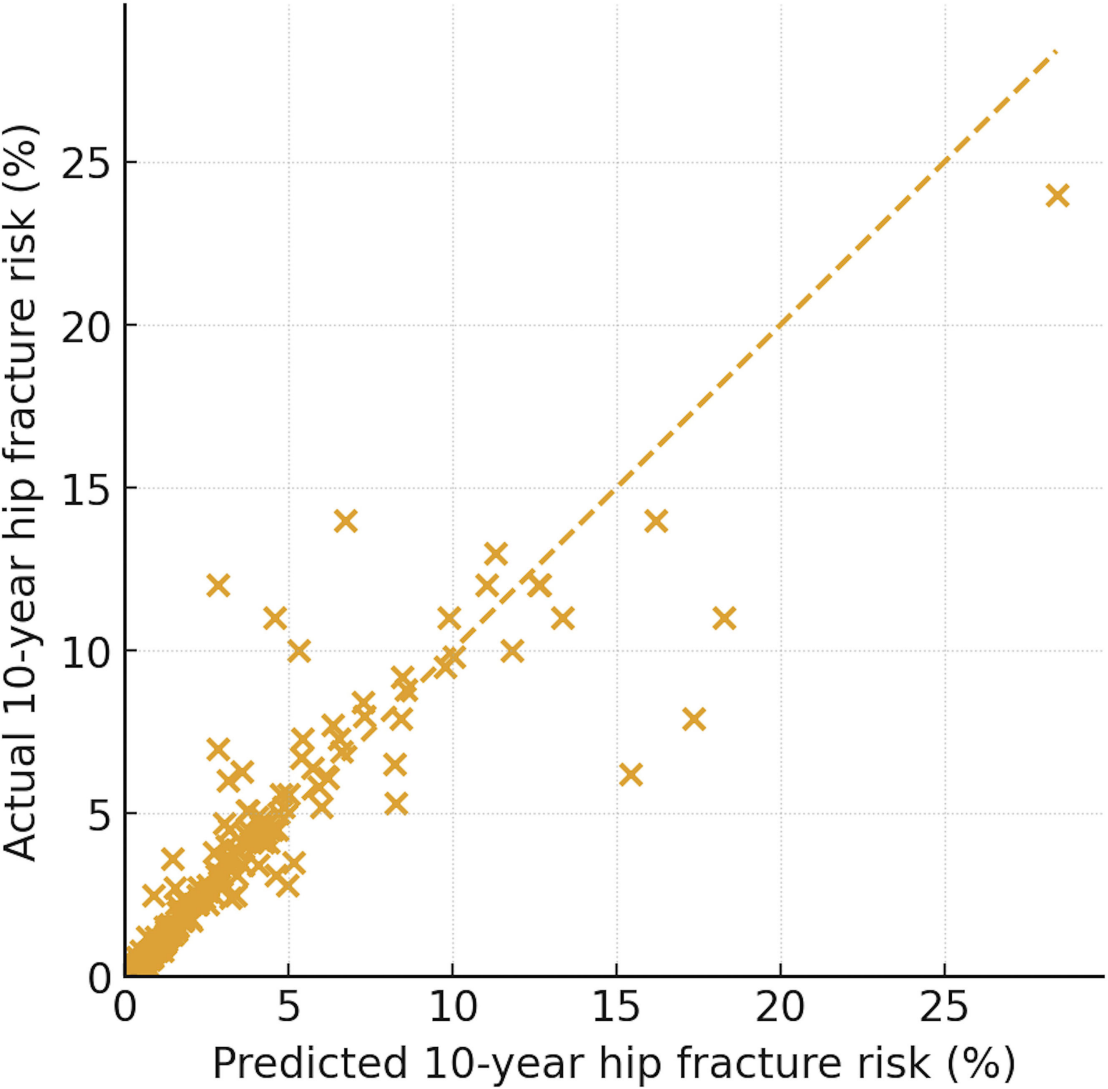
Observed versus model-predicted 10-year FRAX-Hip. Scatterplot comparing the observed 10-year FRAX-Hip with values predicted by the multivariable linear regression model (Model 3). The dashed identity line (y = x) represents perfect calibration. Most points cluster tightly around this line, indicating excellent agreement between observed and predicted risks across the entire range of FRAX-Hip values. Only a few patients with very high FRAX-Hip probabilities are slightly under-predicted by the model. The final model explains approximately 93% of the variance in log-transformed FRAX-Hip (R² ≈ 0.93).

Model 3, which included age, sex, BMI, glucocorticoid exposure, BMD, SDAI, and NLR, explained approximately 93% of the variance in log-transformed FRAX-Hip (R² = 0.932; adjusted R² = 0.930). Diagnostic plots for this model (**Figure 1**) showed no major violations of linear regression assumptions: residuals were randomly scattered around zero without obvious patterns, standardized residuals approximated normality on the Q–Q plot, variance of residuals appeared homogeneous across fitted values, and no highly influential outliers were identified. The scatterplot of observed versus model-predicted FRAX-Hip% (**Figure 2**) demonstrated close alignment of most data points around the identity line, indicating excellent calibration of the linear model over the observed range of hip fracture probabilities.

### Discriminative performance of individual RA activity and CBC-derived indices

**Figure 3:**
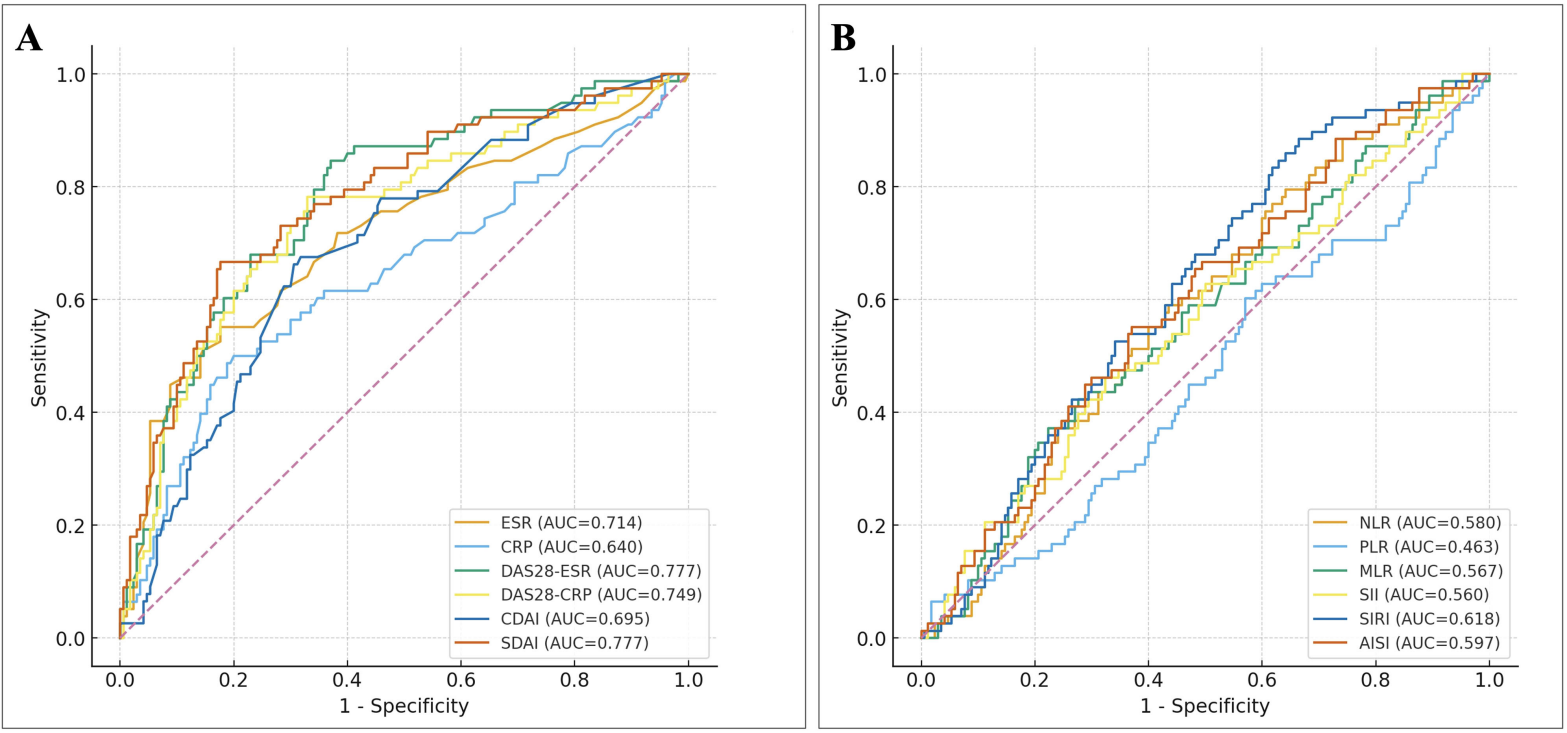
ROC curves of RA disease activity and CBC-derived inflammatory indices for predicting high FRAX hip fracture risk. **(A)** Receiver operating characteristic (ROC) curves comparing the discriminative performance of ESR, CRP, DAS28ESR, DAS28CRP, CDAI, and SDAI for identifying RA patients with high 10year FRAX-Hip fracture risk (FRAXHip ≥3%). AUC values are shown in the legend. SDAI and DAS28ESR achieved the highest AUCs (~0.78), whereas ESR and CRP alone showed only modest discrimination. **(B)** ROC curves for CBCderived inflammatory indices—including NLR, PLR, MLR, SII, SIRI and AISI—for predicting FRAXHip ≥3%. All CBCderived indices demonstrated poor discrimination (AUCs ≈0.46–0.62), indicating limited utility as standalone screening tools for high FRAX hip fracture risk. ESR, erythrocyte sedimentation rate; CRP, C-reactive protein; DAS28, 28-joint Disease Activity Score; CDAI, Clinical Disease Activity Index; SDAI, Simplified Disease Activity Index; NLR, neutrophil–lymphocyte ratio; PLR, platelet–lymphocyte ratio; MLR, monocyte–lymphocyte ratio; SII, systemic immune-inflammation index; SIRI, systemic inflammation response index; AISI, aggregate index of systemic inflammation; ROC, receiver operating characteristic; AUC, area under the curve.

We next evaluated RA disease activity measures and CBC-derived inflammatory indices as stand-alone predictors of high FRAX hip fracture risk (**Figure 3**).

Among RA-related markers, composite activity scores provided the best discrimination. SDAI and DAS28-ESR yielded the highest AUCs (both ≈0.78), followed by DAS28-CRP (AUC ≈0.75), ESR (AUC ≈0.71), and CDAI (AUC ≈0.70). CRP alone showed only modest performance (AUC ≈0.64) (**Figure 3A**). In contrast, all CBC-derived indices showed poor discrimination for FRAX-Hip ≥3%, with AUCs ranging from approximately 0.46 for PLR to 0.62 for SIRI (**Figure 3B**). Notably, SDAI clearly outperformed NLR (AUC ≈0.78 *vs* 0.58), underscoring that integrated assessments of RA disease activity capture hip fracture risk more effectively than simple hematologic ratios.

### Incremental predictive value of inflammatory indices beyond clinical risk factors

The incremental contribution of RA disease activity and CBC-derived indices beyond traditional clinical risk factors were quantified using AUC, continuous NRI, and IDI (**Table 4**).

**Table 4:** Incremental predictive value of inflammatory indices for high 10-year FRAX-Hip fracture risk (FRAX-Hip ≥3%) in RA.

Model comparison	AUC (95% CI)	ΔAUC	NRI	IDI	P (ΔAUC)	Interpretation
Base A: Age + Sex + BMI	0.977 (0.962–0.990)	—	—	—	—	Reference baseline
+ ESR (mm/h)	0.978 (0.964–0.990)	0.001	0.540	0.012	0.699	Minimal, not significant
+ CRP (mg/L)	0.982 (0.969–0.992)	0.004	0.660	0.018	0.318	Small, not significant
+ DAS28-ESR	0.983 (0.969–0.993)	0.005	0.828	0.037	0.237	Slight improvement, NS
+ DAS28-CRP	0.986 (0.974–0.996)	0.009	0.819	0.046	0.066	Modest gain, borderline significance
+ CDAI	0.981 (0.967–0.992)	0.003	0.686	0.019	0.366	Small, not significant
+ SDAI	0.990 (0.980–0.997)	0.013	0.903	0.075	0.015	Largest incremental value, statistically significant but effect size modest
Base C: Age + Sex + BMI + CRP + DAS28-ESR	0.984 (0.971–0.994)	—	—	—	—	
+ NLR	0.985 (0.973–0.994)	0.001	–0.036	0.007	0.562	No meaningful improvement
+ PLR	0.985 (0.972–0.994)	0.001	0.171	0.004	0.563	No meaningful improvement
+ MLR	0.986 (0.974–0.995)	0.001	0.214	0.003	0.185	No meaningful improvement
+ SII	0.985 (0.973–0.994)	0.001	0.070	0.003	0.545	No meaningful improvement
+ SIRI	0.985 (0.973–0.994)	0.001	0.125	0.004	0.564	No meaningful improvement
+ AISI	0.984 (0.972–0.994)	0.000	0.028	0.001	0.762	No meaningful improvement

Base model A includes age, sex, and BMI. Each RA disease activity index (ESR, CRP, DAS28ESR, DAS28CRP, CDAI, SDAI) was added individually to Base model A. Base model C includes age, sex, BMI, CRP, and DAS28ESR; each CBCderived index (NLR, PLR, MLR, SII, SIRI, AISI) was added individually to Base model C. The outcome is FRAXHip ≥3%. AUC = area under the ROC curve; ΔAUC = difference in AUC compared with the corresponding base model. NRI = continuous net reclassification improvement; IDI = integrated discrimination improvement. P values are for ΔAUC based on bootstrap resampling. ESR, erythrocyte sedimentation rate; CRP, C-reactive protein; DAS28-ESR/DAS28-CRP, 28-joint Disease Activity Score using ESR/CRP; CDAI, Clinical Disease Activity Index; SDAI, Simplified Disease Activity Index; NLR, neutrophil–lymphocyte ratio; PLR, platelet–lymphocyte ratio; MLR, monocyte–lymphocyte ratio; SII, systemic immune-inflammation index; SIRI, systemic inflammation response index; AISI, aggregate index of systemic inflammation.

The simple clinical Base Model A (age, sex, BMI) already achieved excellent discrimination for FRAX-Hip ≥3% (AUC = 0.977, 95% CI 0.962–0.990). Adding individual RA disease activity measures to Base Model A produced only small changes in AUC. SDAI yielded the largest improvement (AUC = 0.990; ΔAUC = 0.013; P = 0.015), accompanied by substantial NRI (0.903) and moderate IDI (0.075), suggesting a statistically significant but quantitatively modest gain in risk classification. Other activity measures (ESR, CRP, DAS28-ESR, DAS28-CRP, CDAI) resulted in smaller, non-significant increases in AUC (ΔAUC ≤0.009; all P≥0.066) and relatively limited IDI.

The extended clinical Base Model C, which included age, sex, BMI, CRP, and DAS28-ESR, also showed excellent discrimination (AUC = 0.984, 95% CI 0.971–0.994). Adding any CBC-derived index (NLR, PLR, MLR, SII, SIRI, AISI) to Base Model C led to minimal changes in AUC (ΔAUC = 0.000–0.002; all P≥0.185) and very small NRI/IDI values, indicating negligible incremental predictive value.

**Figure 4:**
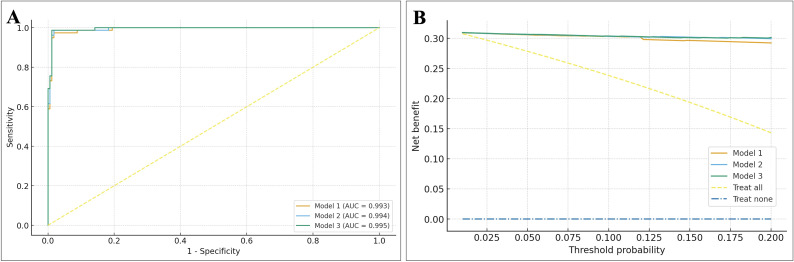
Discrimination and clinical utility of multivariable models for high FRAX hip fracture risk. **(A)** ROC curves for three multivariable logistic regression models predicting high FRAX hip fracture risk (FRAXHip ≥3%): Model 1 (clinical model including age, sex, BMI, glucocorticoid use and BMD), Model 2 (Model 1 + SDAI) and Model 3 (Model 2 + NLR). The clinical base model already shows excellent discrimination (AUC ≈0.99), and adding SDAI or NLR yields only minimal, nonsignificant increases in AUC. **(B)** Decision curve analysis comparing the net clinical benefit of Models 1–3 across threshold probabilities from 1% to 20%. All three models provide higher net benefit than “treat all” or “treat none” strategies. Model 2 and Model 3 show only marginal netbenefit gains over Model 1, indicating that RA disease activity and CBCderived indices add little incremental clinical utility once key clinical predictors are included. BMI, body mass index; BMD, bone mineral density; SDAI, Simplified Disease Activity Index; NLR, neutrophil–lymphocyte ratio; ROC, receiver operating characteristic; AUC, area under the curve.

Consistent with these findings, the full multivariable logistic models incorporating SDAI and NLR (Models 2 and 3) performed almost identically to the purely clinical model (Model 1) in ROC and decision curve analyses (**Figure 4**). AUCs were 0.993, 0.994, and 0.995 for Models 1, 2, and 3, respectively, with virtually overlapping ROC curves. Decision curve analysis showed that all three models provided greater net benefit than “treat-all” or “treat-none” strategies across threshold probabilities from 1% to 20%; however, Models 2 and 3 offered only marginal net-benefit gains over Model 1. Overall, these results indicate that in RA patients, 10-year FRAX-hip fracture risk is driven predominantly by age, sex, BMI, and glucocorticoid exposure, with RA disease activity providing limited incremental improvement and CBC-derived inflammatory indices contributing essentially no additional predictive value once traditional clinical risk factors are considered.

## Discussion

### Key findings and context

In this cross-sectional study of 248 rheumatoid arthritis (RA) patients, we identified age, female sex, low body mass index (BMI), and glucocorticoid use as the dominant predictors of an elevated 10-year hip fracture risk as estimated by FRAX (FRAX-Hip). Importantly, FRAX-Hip is a calculated 10-year probability rather than an observed fracture outcome in our cross-sectional cohort. Notably, these factors are well-established drivers of osteoporotic fractures in both the general population and RA (21). RA confers roughly a two-fold or higher increase in fracture risk compared to non-RA individuals. A meta-analysis reported that RA patients have significantly higher risk of both vertebral fractures (RR ~2.9) and hip fractures (RR ~2.4) than controls (1). The FRAX algorithm was developed to quantify fracture probability based on clinical risk factors, and it appropriately incorporates RA (as a dichotomous yes/no variable) and prolonged glucocorticoid (GC) use as independent risk inputs. Our findings reinforce that these traditional risk factors largely explain variability in FRAX-estimated hip fracture risk among RA patients. In fact, a simple multivariable model including age, sex, BMI, GC use, and bone mineral density (BMD) accounted for over 93% of the variance in log (FRAX-Hip%) in our cohort, with excellent calibration. Accordingly, the very high AUC values observed for clinical models are expected given overlap between the predictors and the FRAX-derived outcome. Practically, this validates the use of FRAX-based risk assessment in RA, especially when BMD is included. Consistent with this, adding BMD measurements can significantly alter risk stratification in RA patients – for example, one study found that incorporating femoral neck BMD into FRAX reclassified about 21% of patients from low to high risk (increasing the proportion of high-risk patients from 25% to 33%) (22). At the commonly accepted intervention threshold of FRAX-Hip ≥3% (10-year risk) (23), our data show that a substantial subset of RA patients would be considered high risk, highlighting the importance of routine fracture risk evaluation in this population.

### RA disease activity and FRAX risk

We explored whether RA-specific disease activity indices provide additional predictive value for fracture risk beyond the traditional factors. Disease activity in RA has long been implicated in osteoporosis and fracture pathogenesis. Chronic inflammation drives systemic bone loss through cytokine-mediated osteoclast activation, and clinical factors like longer RA duration, higher disease severity, and disability have been associated with lower BMD and higher fracture incidence (21). In our cohort, higher RA activity (especially as measured by the Simplified Disease Activity Index, SDAI) was indeed associated with higher FRAX-Hip percentages. Patients with active, uncontrolled RA tended to have elevated fracture risk estimates, whereas those in remission or low disease activity had lower FRAX scores. These observations align with the concept that cumulative inflammatory burden contributes to skeletal fragility. For instance, Phuan-Udom et al. reported that RA patients with higher cumulative DAS28 disease activity had significantly greater 10-year FRAX fracture probabilities, and that moderate-to-high FRAX scores were common in those with persistently active disease (8). Similarly, a large prospective study in early RA found that patients who achieved sustained remission (DAS28 ≤2.6 over the first 2 years) had a markedly lower incidence of fragility fractures, whereas those with persistently high DAS28 scores had increased fracture rates independent of baseline BMD (24). In our cross-sectional analysis, however, the incremental predictive value of incorporating RA disease activity indices on top of the baseline FRAX model was only modest. When age, sex, BMI, GC exposure, and BMD were already accounted for, adding a disease activity measure improved risk discrimination only slightly. This suggests that while active RA is epidemiologically linked to fractures, much of that risk is mediated through factors already captured by FRAX (such as GC use, disability-related low BMI, or already low BMD). Moreover, FRAX already assigns a generic elevated risk for the presence of RA, which may implicitly represent the average impact of RA-related inflammation on fracture risk. Our data indicate that refining this risk by plugging in an exact disease activity score yields limited gain in accuracy for 10-year hip fracture prediction.

Importantly, among the RA activity measures we tested (SDAI, Clinical Disease Activity Index [CDAI], and 28-joint Disease Activity Score [DAS28]), the SDAI stood out as the strongest predictor of higher FRAX-Hip risk. SDAI showed better discrimination and net reclassification of high-risk patients than DAS28 or CDAI. There are a few possible explanations for SDAI’s superior performance. First, SDAI includes a direct measure of inflammation (C-reactive protein) and combines physician and patient global assessments with joint counts, potentially capturing the true disease activity more comprehensively. In contrast, DAS28 (especially DAS28-ESR) can sometimes misclassify patients as in remission despite residual inflammation in joints not counted (e.g. feet) or due to its formula weighting (25). Indeed, the DAS28 remission criterion is known to be less stringent than SDAI remission, meaning DAS28 may label some patients as low disease activity while SDAI still detects active disease. This could make DAS28 a slightly noisier indicator in relation to systemic outcomes like fracture risk. Second, SDAI and CDAI use a 28-joint count similar to DAS28, but SDAI’s inclusion of CRP likely makes it more sensitive to subclinical inflammation that might contribute to ongoing bone loss. Our findings resonate with clinical intuition and prior observations that effective control of inflammation is beneficial for bone health. For example, Yoshii et al. showed that RA patients who sustained remission according to SDAI criteria had significantly fewer new fragility fractures over time (24). Achieving SDAI-defined remission or low disease activity could therefore be a meaningful clinical goal not just for joint health but also for reducing long-term fracture risk. Nonetheless, it must be acknowledged that even SDAI’s added prognostic value in our study was modest – improving the area under the ROC curve only marginally – which tempers enthusiasm for routinely integrating disease activity scores into fracture risk calculators. It appears that extreme levels of disease activity (e.g. uncontrolled inflammation over years) are required to appreciably elevate fracture risk beyond what is already flagged by traditional factors.

### Limited utility of hematologic inflammatory markers

Another question we addressed was whether systemic inflammatory markers derived from the complete blood count – such as the neutrophil–lymphocyte ratio (NLR), platelet–lymphocyte ratio (PLR), monocyte–lymphocyte ratio (MLR), or composite indices like the systemic immune-inflammation index (SII), systemic inflammation response index (SIRI), and aggregate index of systemic inflammation (AISI) – could serve as easily measurable predictors of fracture risk in RA. These CBC-derived indices have attracted interest as potential biomarkers in various diseases, and some studies have linked elevated NLR or related indices with osteoporosis or fracture outcomes. For example, Song et al. reported that postmenopausal women with RA who had higher NLR, PLR, or MLR were more likely to have low BMD and had increased incidence of vertebral fractures on follow-up (hazard ratios ~2–5 for high *vs*. low NLR) (26). The proposed rationale is that a higher inflammatory cell ratio reflects a pro-inflammatory state that might coincide with greater osteoclast activation or frailty. However, in our dataset these markers showed poor ability to discriminate high FRAX-Hip risk. The AUCs for NLR, PLR, MLR, SII, SIRI, or AISI in identifying patients above the 3% FRAX-Hip threshold were only in the 0.46–0.62 range (near random chance for some indices). Even when added to multivariable models, they failed to improve predictive performance in any meaningful way. Several reasons could explain this lack of utility. First, these indices are non-specific and can be influenced by a myriad of acute factors (infections, stress, medications) that are unrelated to the chronic bone remodeling processes underlying osteoporotic fracture risk. In addition, our CBC-derived indices were assessed at a single time point and may fluctuate with transient conditions, further limiting their prognostic stability. In an RA population, patients often have fluctuating blood counts due to disease flares or treatments, which may blur any stable association with long-term fracture risk. Second, any correlation between inflammatory cell counts and fracture risk might be mediated by the same factors we already measure: for instance, severe RA inflammation will raise NLR but also likely leads to higher GC usage, lower physical activity, and more bone loss – all of which are captured by FRAX (through the GC variable, disability-related BMI loss, or via low BMD). Thus, once those main factors are accounted for, an elevated NLR may not carry independent information. Notably, CBC-derived indices may still have value in disease-specific diagnostic contexts (e.g., sacroiliitis in familial Mediterranean fever) (27), supporting the notion that their clinical utility can be condition- and setting-specific. Our findings therefore do not support the use of these blood-based indices for routine fracture risk stratification in RA. They add complexity without clinical benefit, at least in cross-sectional risk estimation. This is in line with the broader literature questioning the specificity of NLR/PLR as prognostic tools for individual patients. Until more consistent evidence emerges, clinicians should focus on direct measurements of bone health and traditional risk factors rather than surrogate blood markers.

### Clinical implications for risk assessment and fracture prevention in RA

Our results have practical implications for improving bone health management in RA. Firstly, they reinforce that RA patients – especially older women, those with low BMI, and those on chronic steroids – should be proactively evaluated for osteoporosis and fracture risk. These high-risk features mirror the general osteoporosis population, but their convergence in RA is common and often compounded by the disease itself. FRAX (with BMD inclusion whenever possible) appears to be a valid and very useful tool in this setting. We observed excellent calibration of FRAX-predicted probabilities to our RA sample’s risk factors, which aligns with a recent large validation study showing that FRAX performs reasonably well in contemporary RA patients (28). In that study, FRAX slightly overestimated risk in the highest-risk RA subgroup but was overall well calibrated, and its risk stratification ability, while slightly attenuated in RA versus non-RA, remained robust. This suggests that clinicians can generally trust FRAX outputs for guiding therapy decisions in RA, with the caveat that FRAX does not explicitly adjust for very aggressive disease phenotypes. Given that, a prudent approach is to use FRAX as a baseline and also consider clinical judgment for extreme cases (e.g. a young RA patient with highly active disease and rapid bone loss might warrant intervention even if FRAX is still below threshold).

Our finding that the Simplified Disease Activity Index (SDAI) (among disease activity scores) best correlates with fracture risk highlights the importance of tight disease control. While adding SDAI to risk models only modestly improved prediction, achieving sustained remission (e.g., early sustained DAS28 remission) has been associated with lower fracture risk in observational studies (24). Whether cumulative or sustained high SDAI over time independently predicts incident fractures beyond FRAX warrants prospective validation. Therefore, aggressive treat-to-target RA management may confer skeletal benefits over the long term, complementing direct osteoporosis therapies. This reinforces current EULAR and ACR recommendations that RA disease activity be tightly controlled not only to prevent joint damage but also to reduce comorbidities like osteoporosis. In practice, rheumatologists should ensure that patients with high inflammation are counseled and monitored for bone density loss. Interdisciplinary care with endocrinologists or primary care for bone health may be warranted in severe RA cases.

Another key implication is the pivotal role of BMD measurement. In RA patients with risk factors, performing a DEXA scan is critical – FRAX without BMD can underestimate risk in individuals who have significant RA-related bone loss. As mentioned, adding BMD can reclassify a substantial proportion of patients into higher risk categories (22), which can change management (e.g. triggering osteoporosis treatment). Moreover, our data and prior studies indicate that many RA patients at high fracture risk are not receiving osteoporosis medications. This treatment gap needs to be addressed. Rheumatology clinics could implement routine FRAX (with DEXA) assessments for patients over a certain age or on prolonged steroids and have protocols for starting calcium/vitamin D and anti-osteoporotic drugs (bisphosphonates, etc.) when FRAX exceeds thresholds or when osteoporosis is present. It is worth noting that current guidelines (such as the NOF and ACR guidelines) consider a FRAX 10-year hip fracture probability ≥3% (or major osteoporotic fracture ≥20%) as an indication for pharmacologic osteoporosis therapy (23). Applying these thresholds to RA patients – who may not traditionally be screened as aggressively as postmenopausal women – could substantially reduce fracture incidence if followed. Our study supports using the 3% hip risk benchmark in RA to identify those who should be treated or receive further evaluation.

On the other hand, the lack of added value from the hematologic inflammation indices suggests we should not rely on surrogate blood tests to estimate bone risk. A normal NLR or platelet count, for example, should not give false reassurance if the patient has other risk factors. Conversely, an elevated NLR in an RA patient should prompt an evaluation for active disease or infection, but it does not specifically mandate bone protection measures unless standard risk factors are present.

### Strengths and limitations

This study’s strengths include a well-characterized RA cohort with comprehensive clinical, laboratory, and BMD data, enabling direct comparison of traditional and RA-specific predictors of FRAX-Hip risk. We applied robust statistical models, including penalized logistic regression and reclassification metrics, to quantify the incremental value of disease activity and inflammatory indices. The use of FRAX-Hip ≥3% as a validated clinical threshold enhances relevance.

However, limitations include the cross-sectional design using FRAX-estimated rather than observed fracture outcomes. The sample was from a single center, limiting generalizability. CBC-derived indices were measured at a single time point and may be influenced by transient factors (e.g., stress, intercurrent infection, or short-term disease fluctuations). We focused on FRAX-Hip and did not perform a parallel analysis for FRAX major osteoporotic fracture probability (MOF). Radiographic damage, cumulative steroid dose, and functional status were not included. Lastly, the high performance of the clinical model may partly reflect overlap with FRAX input variables, limiting the marginal impact of added predictors. Longitudinal studies are needed to confirm these findings and guide RA-specific FRAX refinements.

### Future directions

Prospective studies are needed to validate whether RA disease activity—particularly sustained high SDAI—predicts incident fractures independently of FRAX. Future research should explore whether RA-specific FRAX adjustments (e.g., incorporating cumulative inflammation or steroid dose) improve risk prediction. Developing RA-tailored fracture models that integrate functional status, fall risk, and imaging biomarkers may further enhance accuracy. Larger multicenter cohorts should assess fracture risk across diverse RA populations. The poor performance of CBC-derived indices also underscores the need for better biomarkers of skeletal fragility in RA. Until then, clinicians should prioritize traditional risk factors, FRAX with BMD, and tight RA disease control to optimize fracture prevention strategies.

## Conclusion

In this well-characterized RA cohort, 10-year FRAX-Hip fracture risk was driven predominantly by traditional clinical factors—age, female sex, low BMI, and glucocorticoid exposure—with femoral neck BMD further refining risk estimates. Although higher RA disease activity was associated with elevated FRAX-Hip%, its incremental predictive value beyond these established factors was modest, with SDAI providing only small improvements in discrimination and reclassification. CBC-derived inflammatory indices demonstrated poor performance and offered no meaningful contribution to risk prediction. Overall, FRAX with BMD remains a robust tool for identifying RA patients at high hip fracture risk, while optimal fracture prevention should continue to emphasize aggressive control of inflammation, minimization of glucocorticoid exposure, and proactive osteoporosis management.

## Data Availability

The original contributions presented in the study are included in the article/supplementary material. Further inquiries can be directed to the corresponding authors.
